# From delivery to identity: applying transaction cost economics to health-enabled civil registration in India

**DOI:** 10.3389/fpubh.2026.1743341

**Published:** 2026-02-17

**Authors:** Sheetal Verma, Ritul Kamal, Laxmi Kant Dwivedi, Shiva S. Halli

**Affiliations:** 1 International Institute for Population Sciences, Mumbai, Maharashtra, India; 2 Indian Administrative Service, Lucknow, Uttar Pradesh, India; 3 CSIR-Indian Institute of Toxicology Research (CSIR-IITR), Lucknow, Uttar Pradesh, India; 4 International Institute for Population Sciences, Mumbai, Maharashtra, India; 5 Rady Faculty of Health Sciences, College of Community and Global Health, University of Manitoba, Winnipeg, MB, Canada

**Keywords:** civil registration and vital statistics, heath institutions, key-informant interviews, sustainable development goal, transaction cost economics, Uttar Pradesh

## Abstract

**Introduction:**

This study demonstrates that achieving Sustainable Development Goal (SDG) 16.9—universal legal identity through timely birth and death registration necessitates institutional redesign, best analyzed through the framework of Transaction Cost Economics (TCE). In India, civil registration faces high, uneven transaction costs—travel, documentation, procedural complexity, and information gaps—that disproportionately affect marginalized groups. TCE explains how current systems shift the registration burden onto individuals instead of leveraging health facilities as accessible service platforms.

**Methods:**

The study employed a mixed-methods approach combining conceptual inquiry, policy analysis and field data collection using key-informant interviews in Uttar Pradesh using three separate modules for general public, registrars and executive magistrates, with an aim to capture the diverse perspectives on the barriers of timely birth and death registration.

**Results:**

Drawing on data collected the study shows that delays in birth and death registration were driven by systemic barriers and transaction costs rather than disinterest. Among 142 general public respondents, 55% registered within 21 days, while 20% delayed over 30 days mainly due to low awareness (81%), absent hospital certificates (30%), and access challenges (20%). Timeliness of registration improved in health facilities (~80%) with Auxiliary Nurse Midwives (ANM)/Accredited Social Health Activists (ASHA) support. Registrars and magistrates reported workload pressures, verification challenges, and inconsistent procedures, underscoring the need for institutional reforms to streamline registration in case of delayed registration under section 13(3) of the Registration of Births and Deaths (RBD) Act.

**Discussion:**

The results of the study highlight that integrating civil registration into health facilities can reduce transaction costs, improve equity, and ensure timely coverage. Health institutions are accessible, trusted, and already collect essential data. Supported by digital infrastructure and clear roles, proactive, health-based registration offers a scalable, cost-effective, and inclusive strategy for universal legal identity and strengthened governance.

## 1 Introduction

The global consensus around Sustainable Development Goal (SDG) 16 emphasizes not only peace and justice but also the construction of institutions that are inclusive, accountable and efficient. Within this expansive vision, SDG Target 16.9—ensuring legal identity for all, including birth registration, represents a foundational aspiration for equitable governance and citizen inclusion (1). Legal identity is not merely a symbolic right but the gateway to exercising all other rights: accessing healthcare, enrolling in school, claiming social protection, participating in elections and inheriting property (1, 2). Yet for millions across the Global South, including a significant segment of India’s population, access to civil registration remains obstructed by institutional fragility, infrastructural shortfalls and administrative bottlenecks (3–6). One of the most persistent barriers is the incomplete or delayed registration of births and deaths, which undermines the foundation of inclusive identity systems. According to the latest, Civil Registration System (CRS) report 2022, although national-level registration coverage has increased substantially, yet only around ~74.4% of births and ~73.8% of deaths are registered within the legally mandated 21-day period, with wide variations in the registration levels among the states and union territories (3). The remaining unregistered cases amounting to several 100,000 births and deaths pose significant challenges. These delays contribute to disparities in death registration, regional imbalances and systematic under-registration among disadvantaged groups. More importantly, they render individuals temporarily invisible to the state, cutting them off from essential services and protections. This is not merely a problem of low awareness or deliberate neglect; rather, it reflects deeper systemic inefficiencies in how registration services are designed, delivered and embedded in the everyday experience of state–citizen engagement.

This paper is motivated by the recognition that transaction costs, the often-invisible burdens of information seeking, travel, paperwork and enforcement are a central, though not exhaustive, factor contributing to the civil registration gap. These costs are unevenly distributed, falling most heavily on disadvantaged populations: those living far from registration offices, women and children with limited access to state institutions and families unfamiliar with bureaucratic procedures. However, individuals do not make decisions based on costs alone; they weigh these against perceived benefits. A key insight is that the benefits of civil registration especially in India often appear distant, uncertain, or intangible, such as future access to social protection or legal identity, making it less likely that they outweigh the immediate and visible transaction costs.

### 1.1 Transaction cost economics

In this context, the research proposes the application of Transaction Cost Economics (TCE)—an institutional economics framework developed by Coase (7) and Oliver Williamson (8) as a useful lens to diagnose and reform India’s Civil Registration and Vital Statistics (CRVS) system. TCE redirects attention from presumed motivational deficits among citizens to structural inefficiencies in service delivery. It asks: *under what institutional arrangements can the cost of a transaction—such as registering a birth or death be minimized?* and *how can the state design systems that reduce friction, duplication and barriers, while also making the future benefits of registration more visible and accessible to those currently excluded?*

The theory of Transaction Cost Economics offers a compelling lens for examining how public services are structured, accessed and delivered particularly in complex sectors like healthcare and civil registration, initially developed by Coase (7) and later expanded by Williamson (9, 10). TCE begins with a deceptively simple insight: markets are not costless. Every transaction whether economic, legal, or administrative entails costs beyond the price of the good or service itself, which include the costs of searching for information, negotiating agreements, enforcing compliance and monitoring execution. Institutions, whether firms, government agencies, or hybrid arrangements exist because they reduce these transaction costs more effectively than uncoordinated markets or decentralized action. While originally developed in the context of private firms deciding between in-house production and outsourcing, TCE has been fruitfully applied to various domains of public administration, especially in health systems, where the boundaries between providers, clients and regulators are often blurred and where uncertainty, asset specificity and asymmetry of information are high.

In the health sector, TCE helps explain why certain services—such as disease surveillance, immunization and maternal care are better delivered through vertically integrated public institutions than through loosely coordinated private actors or fragmented NGOs (8) Health services involve high levels of uncertainty (about need, effectiveness and risk), asset specificity (specialized training, equipment and trust) and coordination intensity (among patients, families, providers and payers), which make purely market-based delivery not only inefficient but potentially harmful, especially for poor and vulnerable populations (10). Scholars such as Burns et al. (11) and Roberts et al. (12) have applied the TCE framework to explain government preferences for integrated service delivery over outsourcing in primary care and public health. Their work shows that in high-transaction-cost environments, such as rural India or remote areas, internalized service provision through health systems is not only more equitable but also more cost-effective in the long run.

When applied to the interface between health systems and civil registration, TCE reveals a similar logic. The act of registering a birth or death, especially in low-income, remote, or marginalized settings is not a simple administrative formality, but is a cost-intensive process involving documentation, navigating external costs and repeated visits. All these transaction costs burden the households, who are often with limited resources and finally end up in families delaying registration as the benefits of registration are often delayed or indirect. This behavior is not irrationality or ignorance, but an economically rational response to a high-cost, low-trust transaction system.

From the perspective of the state, these user-side transaction costs translate into broader administrative and governance costs. Unregistered births and deaths lead to incomplete demographic data, poor targeting of services and ineffective monitoring of development goals, while the retrospective registration drives and data reconciliation result in higher costs than timely registration Therefore, as per the theory of TCE, when external costs are high, internalization becomes the efficient choice. In India, health facilities can function as such institutions are they already collect, verify and report birth and death data. Health facility staff including medical officers, ASHAs, ANMs are trained in documentation and reporting and also record births and neonatal deaths through HMIS in many states. Extending their mandate to include legal registration, supported by digital tools such as mobile/API integration, entails relatively lower registration costs while yielding substantial savings in transaction costs for the citizens.

In most states of the country this model is already in vogue, with midwives or health officers empowered to notify or even register vital events and private health facilities reporting data directly to local area registrars, enabling faceless registration and issue of certificates before discharge. Studies have shown that such integration improves registration completeness, reduces delay and increases equity, especially among rural and poor populations. In India, similar efforts are visible in most states, for example in the states of Uttar Pradesh, where birth registration has been linked to institutional deliveries and real-time data transmission from government health facilities to civil registration portals is piloted (13–16). However, these remain as exceptions rather than the norm. In majority of the states, lower-level health facilities are passive data sources rather than active registration units, while the private facilities, barring the ones in major cities rarely act as effective informants, resulting in families to initiate and complete registration process at distant registration offices, often with limited accessibility and user-friendly infrastructure.,

TCE would argue that this institutional design is suboptimal. It creates what Williamson calls *“maladaptation costs”* i.e., costs that arise when institutions are not aligned with the nature of the transaction (17). The nature of birth and death registration is personal, localized, time-sensitive and document driven, making it well suited for internalization within the health facilities, however the current design externalizes the burden onto families leading to inefficiencies, delays and under-registration. Moreover, the fragmentation between health and civil registration systems adds coordination costs for the state itself, as the data collected at the time of delivery (e.g., date, location, mother’s age, child’s gender) is often not shared with the registrar, leading to duplication, reconciliation errors and data gaps. TCE predicts that such misalignment increases the total cost of service delivery, even if each agency is doing its own job efficiently.

One of the central insights of TCE is that institutions should be designed not just to deliver services, but to reduce the cost of delivering services, both for the provider and the user. Applied to the case of CRVS this implies reducing ex-ante transaction costs (searching for information, locating offices), the ex-post costs (correcting errors, making follow-up visits) and the enforcement costs (ensuring compliance through legal mandates or incentives). Health facilities, when legally designated and technologically enabled, can help achieve all three, e.g., Primary Health Centres (PHCs) and Community Health Centres (CHCs) embedded in the local communities, follow standardized data workflows and serve as primary points of contact at critical life events. By embedding registration into these moments, the state can move toward a proactive, rather than reactive, model of legal identity provision.

In addition, the notion of asset specificity, a key concept in TCE, applies well to the health–CRVS connection. Health workers possess context-specific knowledge, community trust and specialized training that make them uniquely suited to collect and validate vital event data. Asking an unrelated registrar, located far away, to verify a birth that occurred at a distant health facility introduces unnecessary duplication and reduces data quality. Similarly, digital systems like India’s HMIS and the RCH portal already capture vital event data with increasing coverage, quality and reliability. It is known that strategic interventions such as data quality audits, supportive supervision, training at all levels and dashboards for real-time monitoring have improved both the accuracy and use of routine health data for decision making (18). Sharing such high-quality, well-managed data with civil registration systems securely and under proper protocols could enhance completeness, timeliness and reliability of vital event registration. Not leveraging these existing, quality-assured assets represents a missed institutional opportunity to strengthen both health and civil registration systems.

Moreover, TCE’s emphasis on bounded rationality, i.e., the idea that people make decisions based on limited information and cognitive capacity explains why even well-intentioned families may fail to register births or deaths. In high-stakes, high-stress moments like childbirth or bereavement, expecting families to initiate a complex bureaucratic process is unrealistic. Instead, institutions should be designed to function on the principle of *suo-moto* registration—automatic or semi-automatic initiation of legal recognition based on institutional data. This is especially feasible in health settings, where data on the event already exists and can be verified at source. *Suo-moto* registration not only reduces transaction costs but also enhances trust in government, as citizens see the state acting proactively and responsively.

Finally, the principle of economizing on governance costs, central to TCE, has important implications for CRVS reform. By converging health and registration functions, the state can reduce interdepartmental coordination costs, streamline accountability and achieve better outcomes without significant new investments. Instead of creating parallel systems or hiring new staff, the government can repurpose and train existing health workers MOICs–Medical Officer In-Charge, ANMs–Auxiliary Nurse Midwives, ASHAs–Accredited Social Health Activists, Data Entry Operators and even private sector operators to carry out CRVS functions with minimal incremental effort. When combined with digital tools, mobile apps and biometric validation (e.g., Aadhaar-linked birth certificates), the system becomes not only cheaper to run but also more user-friendly, secure and resilient.

In sum, the application of TCE to civil registration through health facilities offers both a theoretical justification and a practical roadmap for reform. It moves the conversation beyond awareness and incentives to the deeper question of institutional design. It explains why current CRVS systems—fragmented, bureaucratic and citizen-initiated are inherently prone to delays and exclusions. The present study applies the TCE framework to examine the delays in birth and death registration. Drawing on primary data from the state of Uttar Pradesh, the study aims to document the barriers to timely registration of births and deaths and to capture the real-world experiences of delay, confusion, multiple visits, document gaps and knowledge asymmetries in the registration process.

## 2 Materials and methods

This study centers its analysis on India’s health facilities, positioning them as pivotal institutions for strengthening the country’s CRVS system. As of March 31, 2023, the public healthcare infrastructure alone comprised 1,69,615 SCs, 31,882 PHCs, 6,359 CHCs, 1,340 Sub-Divisional and District Hospitals (SDHs), 714 District Hospitals (DHs), 362 Medical Colleges (MCs), 23,581 Government Hospitals and 22 Central Government Hospitals including All India Institute for Medical Sciences (AIIMS) (19). These facilities operate within a tiered system, offering services ranging from basic primary care at SCs to advanced tertiary care and medical education at district hospitals and medical colleges.

**Figure 1:**
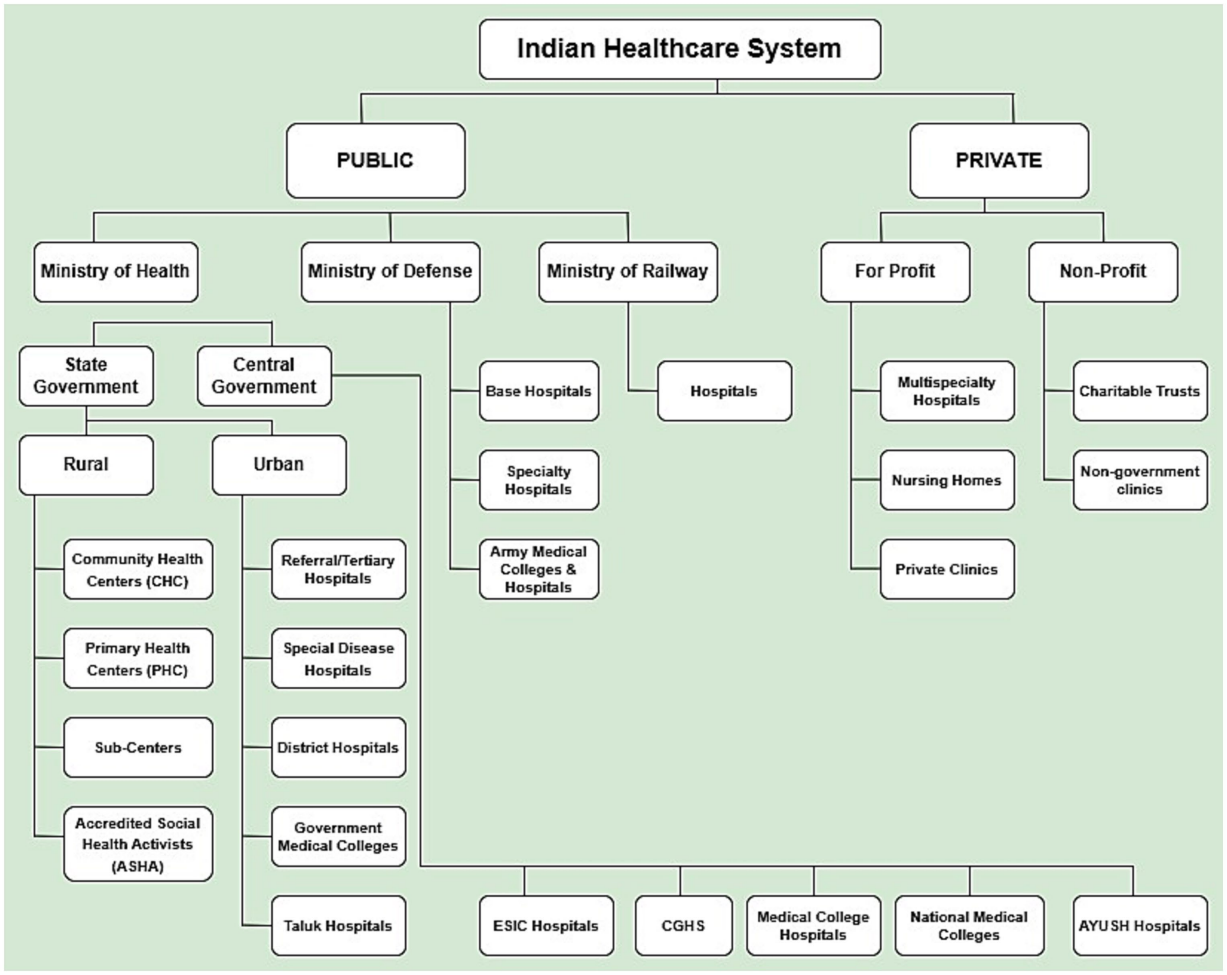
Hierarchy of healthcare system in India.

Over the past two decades, initiatives such as the National Health Mission (NHM) and schemes like Janani Suraksha Yojana (JSY) have significantly expanded the reach and utilization of these health facilities (20, 21). One of the most notable outcomes has been the sharp increase in institutional deliveries, which rose from 40.8% in 2005–06 to 88.6% in 2019–21 (NFHS-5) (22) with several states such as Kerala, Goa and Tamil Nadu achieving near-universal institutional birth coverage (22, 23). Approximately 60% of all institutional deliveries occur in government health facilities, positioning them as logical and accessible nodes for initiating birth registration. These facilities could be formally designated as Registrars, making registration an automatic, integrated process rather than a separate bureaucratic step. Additionally, there are 43,486 private health facilities (24) that, are legally bound to act as informants to local registrars and further streamline the registration process. The hierarchy of the healthcare infrastructure in India has been given in Figure 1.

### 2.1 Study design and participants

To examine the disjunction, the study combines conceptual inquiry, policy analysis and empirical data. Conceptually, we draw on the literature on TCE and its application in public administration, particularly health systems. Methodologically, we adopt a qualitative–quantitative mixed approach. A primary study utilizing household and official responses in several districts of Uttar Pradesh was carried out. The share of timely (within 21 days of the event) birth and death registrations in Uttar Pradesh improved steadily between 2019 and 2022, with birth registration rising from 54.4 to 61.8% and death registration from 52.2 to 68%. However, in 2022, nearly 40% of births and one-third of deaths were still registered with delay (3). By synthesizing the insights from this study with national policy trends and legal developments, particularly the 2023 amendment to India’s Registration of Births and Deaths (RBD) Act—a strong case for institutionalizing civil registration within health facilities as a cost-effective, scalable and inclusive strategy has been made (25).

For the purpose of the study participants from 3 broad group, which play crucial role in the overall birth and death registration process were selected:

1. *General Public:* individuals aged 18 and above and who have registered an event of birth or death
2. *Registrars:* government officials responsible for administering and managing the registrations at local level
3. *Executive Magistrates:* Primarily Sub-Divisional Magistrates (SDMs) and other Executive Magistrates involved in adjudicating delayed registration cases.

The inclusion criteria for the study consisted of any resident of Uttar Pradesh aged 18 years and above who have registered any event of birth or death. It also included registrars and executive magistrate involved in the process of birth or death registration.

### 2.2 Data collection

The study utilized an exploratory mixed-methods approach, with semi-structured key informant interviews (KIIs) were employed as the primary method of data collection. To facilitate this process, three customized questionnaires viz. Registrar Module, Executive Magistrate Module and Public Module were developed and the interviews were conducted either in person or via Google Forms, depending on participant convenience. All the questions were bilingual to facilitate respondent participation and the confidentiality of all respondents were fully adhered. Informed consent was obtained from all the participants before their participation in the study. The study studies involving human participants were reviewed and approved by the Students Research Ethics Committee (SREC) at the International Institute for Population Sciences, Mumbai (Maharashtra, India) (Ref. No. IIPS/AC/SREC/SV/IO-147/2025).

A total of 224 responses were collected across the three modules. Among these, 142 responses were obtained through the Public Module, 59 responses through the Registrar Module and 23 responses through the Executive Magistrate Module.

## 3 Results

### 3.1 Flow of birth and death registration in India

**Figure 2:**
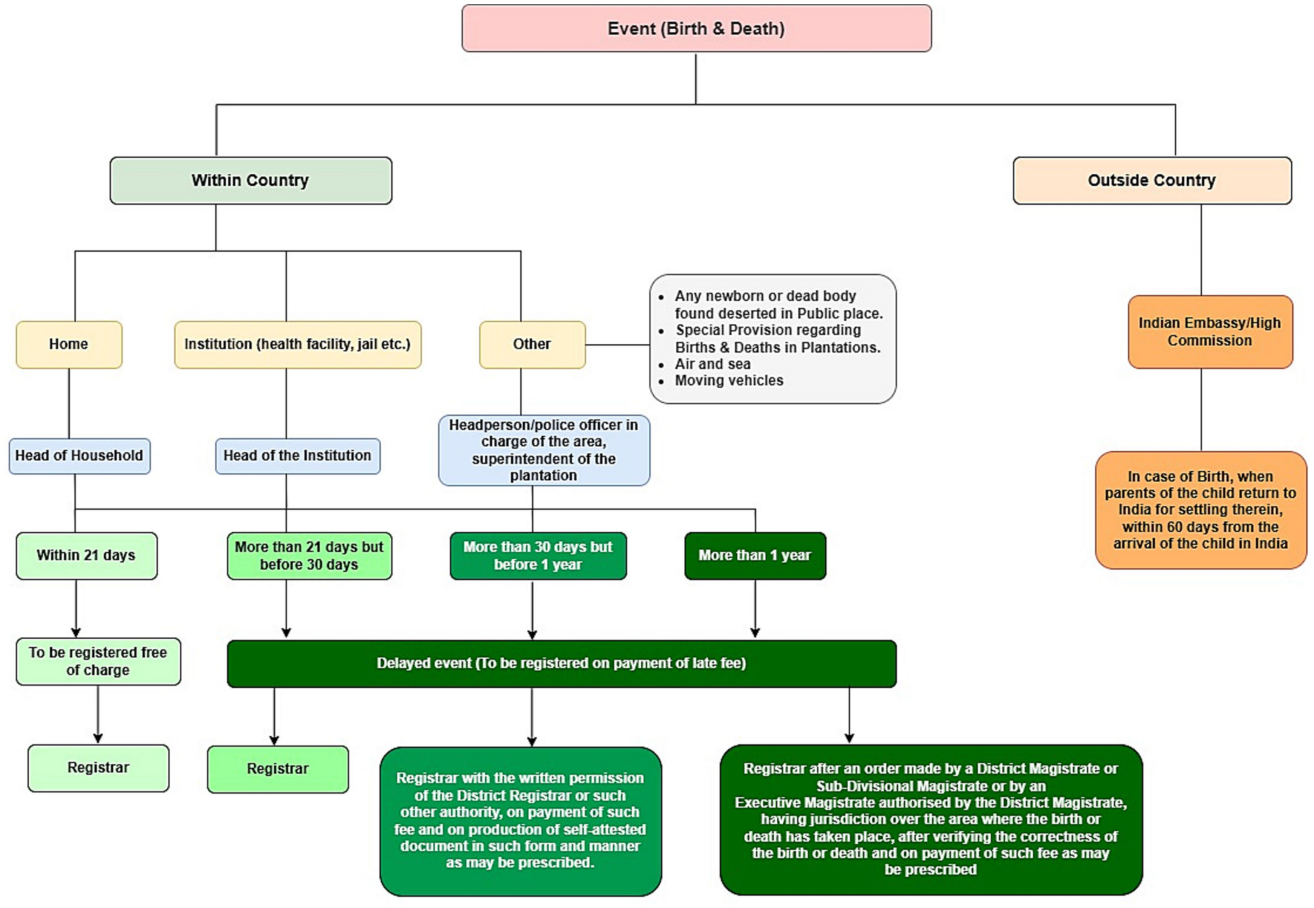
Flowchart of birth and death registration in India.

The flowchart of the birth and death registration process in India, as illustrated, highlights a complex sequence of actors, time-bound procedures and institutional pathways that vary based on the place of occurrence and timing of the event. While the system allows for free registration within 21 days, delays result in increasingly complex bureaucratic steps and additional costs (Figure 2).

### 3.2 Procedure for delayed registration under section 13(3) of RBD act

The manuscript builds on data collected from the general public, registrars, and executive magistrates across multiple districts of Uttar Pradesh, with a focus on delayed birth and death registration. Drawing on inputs from families, health workers, registration officials and Executive Magistrates, the data illustrates how transaction costs within the civil registration system influence both citizen decision-making and institutional functioning. The study targeted three respondent groups critical to understanding delayed registration dynamics. First, Executive Magistrates, typically Sub-Divisional Magistrates (SDMs), who are legally mandated to approve registrations delayed by over 1 year under section 13(3) of the Registration of Births and Deaths (RBD) Act, 1969 and RBD (Amendment) Act, 2023 (25, 26). Second, Registrars and office functionaries who process routine and delayed applications and serve as operational intermediaries in the CRVS framework. Third, members of the general public who had attempted to register births or deaths more than 1 year after the event.

**Figure 3:**
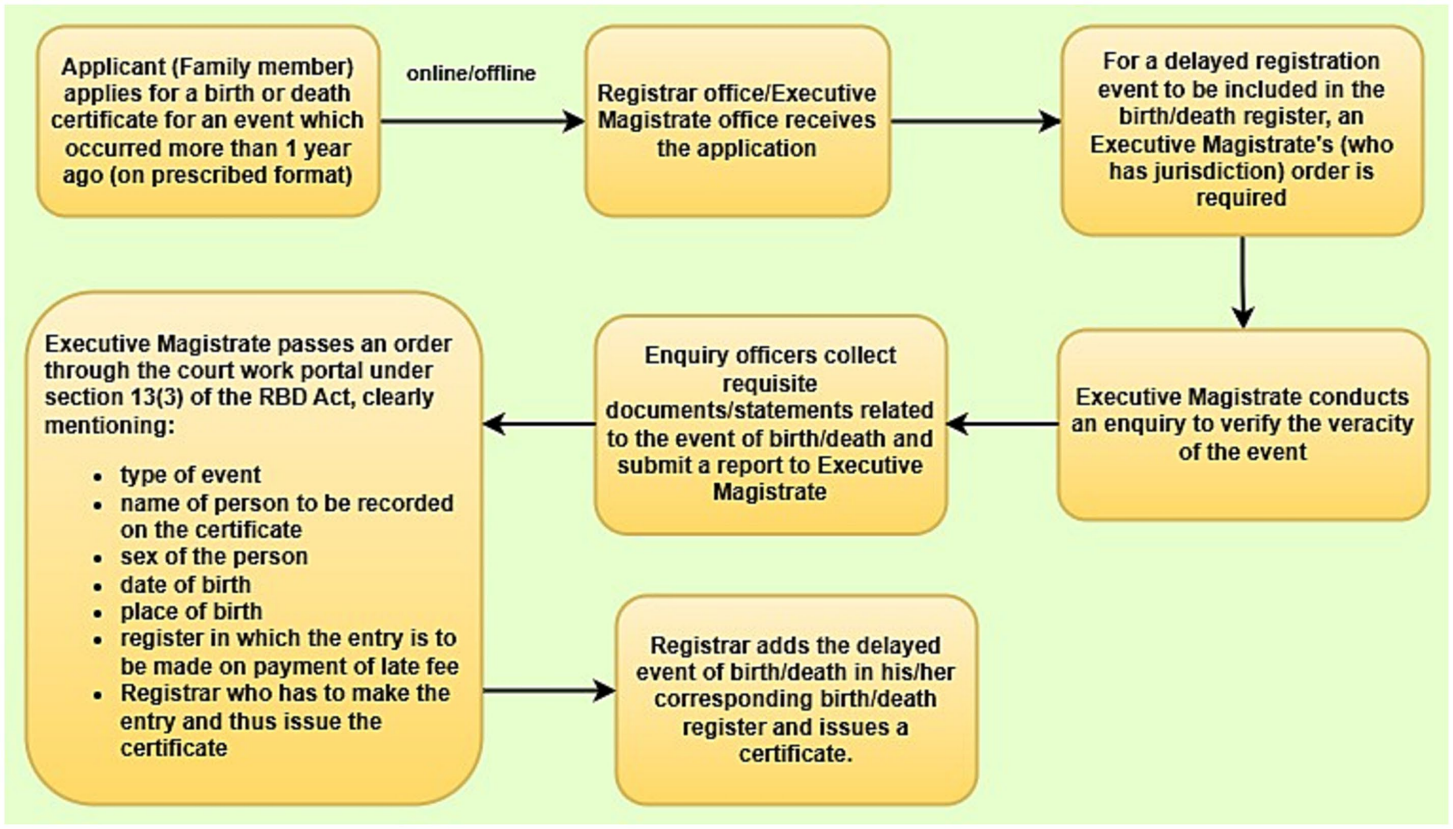
Process chart of delayed registration in India.

Figure 3 below highlights the process chart of delayed registration in India under the provisions of section 13(3) of the RBD Act, 1969 and RBD (Amendment) Act, 2023 (25, 26). The applicant (or a family member) applies either online or offline to the Registrar or Executive Magistrate. For such delayed registrations, an order from the Executive Magistrate is mandatory. The Executive Magistrate initiates an enquiry to verify the veracity of the event, with enquiry officers collecting relevant documents and submitting a report. Based on this, the Magistrate issues an order specifying key details like type of event, name, date and place of birth/death and directs the Registrar to make the entry upon payment of the prescribed late fee. Finally, the Registrar records the delayed event in the register and issues the certificate.

### 3.3 Citizen experiences: awareness, costs, and barriers to timely registration

Out of the 142 respondents, ~89% had registered an event of birth or death, with ~72% being births and ~28% being deaths. Majority of the events occurred in government hospitals (~54%) followed by private hospitals (~23%) and homes (~23%). Respondents reported that Certificates were primarily required for government documents (51.6%), followed by legal purposes (30.3%) and availing benefits under government schemes (32.8%). The results highlight that at the citizen level, one key insight is that the majority of respondents (around 90%) had previously attempted registration and more than half (55%) registered within the 21-day legal window. Yet, a significant share, around 20% reported initiating registration only after 30 days, triggering additional procedures under section 13 of the RBD Act. These differences are crucial: they reveal how institutional proximity and procedural automation reduce transaction costs, while externalized or delayed processes shift the burden to families.

The results revealed that the primary reasons for delay were not disinterest but practical barriers, especially delays and barriers were primarily due to lack of awareness (~81%), difficulty accessing registration units (~20%) and hospitals not providing certificates (~30%). Other challenges included travel costs (~15%), paperwork costs (~14%), need for a lawyer/agent (~7%), lack of priority given by registrar (~11%), and unavailable online registration (~3%). For example, many respondents cited distance to registrar offices, time required and expenses for paperwork and travel as disincentives. The average out-of-pocket expense for delayed registration was estimated between ₹500–₹5,000, with approximately 74% of the respondents’ incurring costs of ₹500, while around 18% spent between ₹500–₹1,000 and around 8% spent up to ₹5,000. These costs were often borne by families themselves. Although this may seem modest, for daily wage earners, women recovering post-delivery, or the older adults, such expenses especially when compounded by uncertainty are sufficient to cause postponement. These frictions align precisely with what TCE defines as ex-ante and ex-post transaction costs, the former referring to search and documentation burdens, the latter to repeat visits, corrections and unpredictable delays.

### 3.4 Role of location of event and frontline workers in reducing transaction costs

Importantly, the site of the event strongly influenced outcomes. Registration was more efficient and timelier when births occurred in health institutions. For events taking place in government hospitals, approximately 80% of respondents either received the birth or death certificate at the time of the event or submitted the application within the mandated 21-day period, thereby avoiding delayed registration. Respondents who received support from registration authorities, ANMs, or ASHAs were significantly less likely to report confusion regarding procedural steps or document requirements. Among those who received such support, approximately 76% reported either obtaining the certificate at the time of birth or death or applying for registration within the mandated 21-day period. These frontline workers play a critical but under-recognized role in reducing informational transaction costs serving as translators of bureaucracy for citizens navigating an opaque system.

This was further reinforced by the finding that online application systems remain underused. Despite digitization efforts, the share of forms submitted to registrars online remains low, due to poor connectivity, digital illiteracy, or lack of access to internet points like cybercafés. Many respondents still prefer (or are forced into) offline, in-person visits, increasing exposure to informal payments, incomplete guidance and bureaucratic gatekeeping. In some cases, middlemen emerged as informal navigators adding further financial cost and eroding trust in the registration system. These dynamics are emblematic of negotiation and enforcement costs in loosely monitored, semi-digital systems, where compliance depends more on interpersonal influence than on institutional clarity.

### 3.5 Supply-side constraints

On the supply side, registration officials offered a strikingly candid assessment of structural limitations. Over 77% were posted in rural units, many of which are understaffed. Most offices reported just 1–2 people managing the entire registration load, handling data entry, verification, public queries and certificate issuance. Though a majority understood the provisions of delayed registration under section 13(3), few had received standardized training. Delays were attributed to incomplete documentation (~78%), challenges in verifying older cases and procedural burdens related to obtaining Executive Magistrate orders (~66%). Additional constraints were time pressures (32.2%), pressure from applicants (47.5%), and technological issues like internet connectivity (27.1%). Delayed registration beyond 1 year was most commonly driven by school or Aadhaar-related requirements for births (84.7 and 83.1%, respectively) and by inheritance, property mutation, or benefit claims for deaths (86.4 and 74.6%). Many registrars highlighted the increased administrative burden from rising public demand for retrospective certificates, particularly for Aadhaar, school admissions, or legal benefits—pressures often applied through local intermediaries or elected representatives.

The volume of delayed registration applications reported also highlights system-level strain. Most registrars process fewer than 100 such applications per month, but some reported dealing with over 500 applications monthly, especially in large or peri-urban blocks. These workloads were not accompanied by proportionate human resources or support infrastructure. Furthermore, registrars described procedural inconsistencies in how applications were processed ranging sending reports to the Executive Magistrate for orders (86.4%), spot verification of events (67.8%), registering based on affidavits/self-declaration (66.1%) and registering based on submitted documents (52.5%). This variation not only causes administrative bottlenecks but also fuels public confusion about what documents are needed and which processes apply.

### 3.6 Executive magistrate’s role and challenges

Responses from Executive Magistrates reinforced the challenges. Most were familiar with delayed registration protocols but described the verification process as time-consuming and logistically demanding. The most commonly cited methods included document scrutiny (78.3%), field verification (78.3%), affidavit or self-declaration by the applicant (73.9%), and Registrar’s report (56.5%). Challenges included difficulty verifying events (73.9%), incomplete documents/affidavits (56.5%), legal pressure from applicants or representatives seeking urgent certificates for school, property, or Aadhaar corrections (39.1%), technological issues like internet connectivity (26.1%), and magistracy workload (43.5%). Some respondents specifically mentioned the need for clearer SOPs, better infrastructure and capacity-building workshops to standardize implementation of section 13(3). One official noted, “When we are doing all the fact-checking and SDM gives the final order based on our report, it would be better to decentralize some authority for older but non-contentious cases.”

### 3.7 Event-specific drivers of delayed registration

The reasons for delayed registration differ by event. For births, the top motivations included school admission, Aadhaar linkage and access to government benefits like scholarships. For deaths, the leading drivers were inheritance and mutation of property records, pensions, insurance claims and legal cases. This reflects the instrumental value of registration, i.e., citizens seek certificates not for their intrinsic value, but because they are gatekeepers to other entitlements. Unfortunately, this reactive model of engagement results in “crisis registrations” rather than routine inclusion, disproportionately burdening families at vulnerable moments. While the majority of the Executive Magistrates were aware about the provisions of delayed registration under section 13(3) of RBD Act 1969/2023, most of the respondents cited lack of training/workshops in the past year.

### 3.8 Demand for health-based registration

Despite these systemic hurdles, there is strong demand for reform. The majority of the public respondents preferred receiving birth or death certificates directly from health facilities, especially during immunization visits or postnatal check-ups. They cited convenience, fewer steps and trusted relationships with health workers. Health staff, particularly ANMs and ASHAs, also expressed support for this model, but stressed the need for formal training, legal backing and digital infrastructure. Currently, most assist informally but are not empowered to complete the process. This gap between informal effort and formal authority mirrors what TCE would characterize as a misalignment of asset specificity—where those best positioned to perform a function are not institutionally recognized for it.

ANMs recognized their potential role but remained unsure about the legal procedures and record-keeping requirements. Medical Officers, meanwhile, admitted uncertainty over whether their responsibilities extended beyond issuing discharge certificates. In effect, legal authorization was present, but institutional clarity and operational preparedness were absent. This points to a deeper governance dilemma: when statutory authority is not backed by adequate infrastructure—human, technological, and procedural—the burden of transaction costs is shifted rather than eliminated.

This underutilization is not only inefficient but also inequitable. Families who deliver in government health facilities, especially first-generation users from remote or marginalized communities are being denied the “benefit of proximity” because of institutional inertia. As shown in survey findings, even when events occur in a health facility, families are often told to return days later to a separate registrar office, sometimes with notarized affidavits, multiple identification proofs and witnesses. These requirements mirror the process for delayed registration under section 13(3), even though the event occurred in an authorized facility. The transaction costs in such cases are not only financial but also psychological and social, particularly for women, older adults caregivers and daily-wage workers who lose time, money and trust in the state. These outcomes are not unintended; they are the direct result of a policy architecture that fails to activate the legal capacities it already possesses.

In terms of cost, responses from officials and families confirmed that the average expense for delayed registration ranges from ₹500–₹5,000, depending on the location, legal requirements and assistance sought. Costs include travel, affidavit notarization, photocopies and occasionally informal facilitation fees. These costs, while modest in absolute terms, represent real burdens for the poor. Worse, most applicants bear these costs themselves, often without support or reimbursement. From a TCE perspective, this indicates a lack of cost-internalization by the system, a critical flaw in public service design.

The data also surfaced deep institutional pressure on registrars and magistrates. Officials frequently reported being pressured by applicants, schools, legal representatives and even elected leaders to expedite registration despite incomplete documentation. These demands are often driven by urgent timelines, i.e., school deadlines, legal hearings, or Aadhaar issues but undermine due process. The perception that procedures are deliberately obstructive leads some citizens to view registrars as adversaries rather than facilitators, further eroding institutional trust. These pressures and the defensive bureaucracy they engender are classic manifestations of what TCE calls enforcement risk in low-trust, high-cost environments.

Taken together, these findings strongly support the TCE proposition that institutional design determines transaction costs and that unless these costs are reduced, civil registration will remain uneven, exclusionary and reactive. The consistent demand for health-based registration, combined with the demonstrated capacity of CHCs and PHCs to record events accurately and on time, makes a compelling case for reform. Health facilities are physically proximate, socially embedded and already engaged at the point of event. If trained, equipped and legally authorized, they can serve as effective registration nodes reducing transaction costs for families and administrative burdens for the state.

The TCE framework also suggests that uncertainty about procedures, requirements, or outcomes increases the transaction cost of public services. The results confirm that many families are uncertain about how to register a birth, what documents are required, or what deadlines apply. Health workers, in contrast, have standardized protocols and greater procedural certainty. If empowered to complete or initiate registration on behalf of families, they can absorb this uncertainty, reducing the burden on citizens and increasing overall compliance. From a governance perspective, this reduces the need for penalties, awareness campaigns and retrospective corrections all of which carry their own costs.

**Figure 4:**
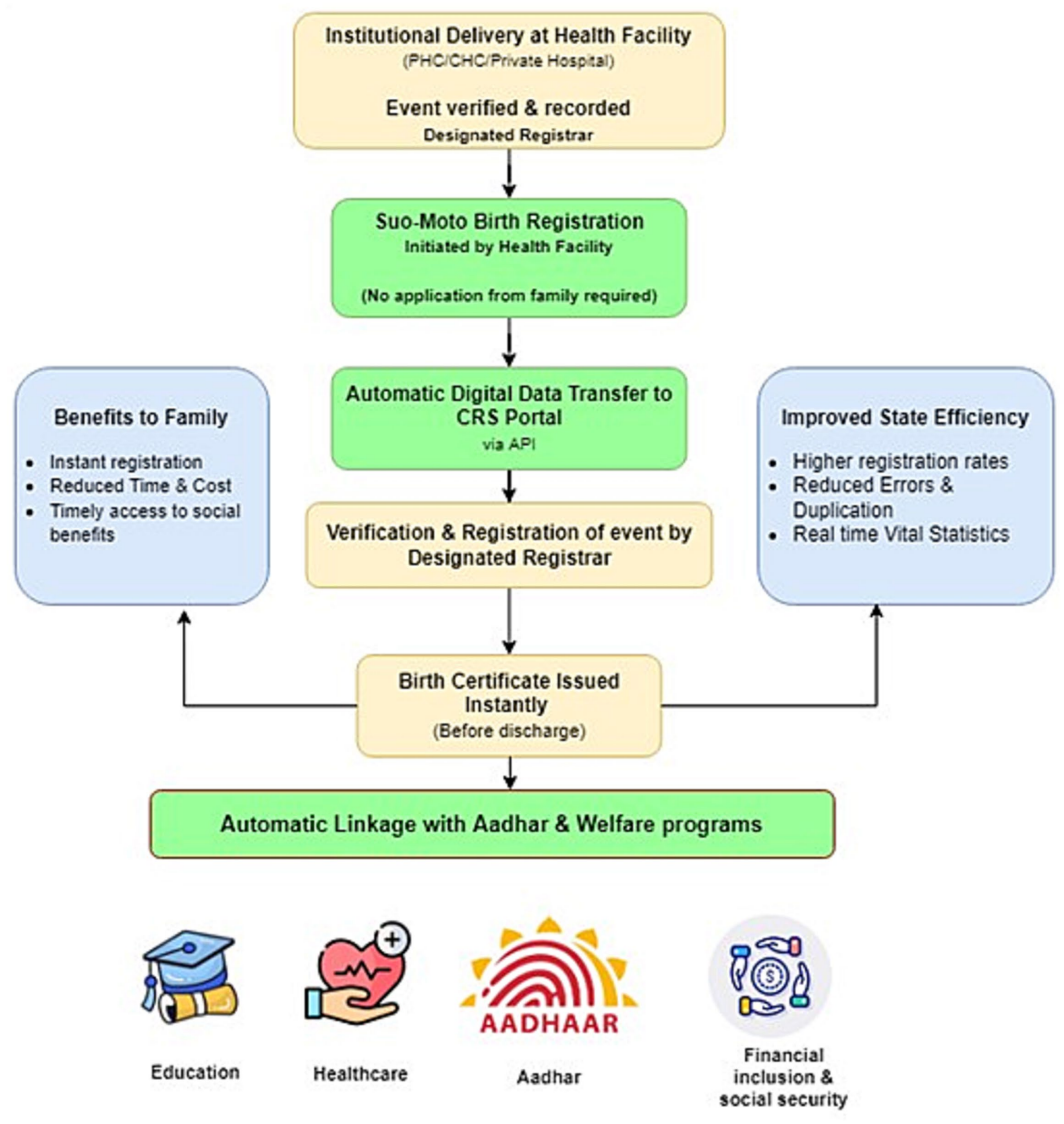
Proposed flowchart of birth and death registration in India, integration registration within healthcare institutions.

Figure 4 presents a proposed framework for birth and death registration in India, integrating the process within the existing healthcare system. Vital events are verified at health facilities and registered suo-moto, with automatic digital transfer to the CRVS portal and back-end verification by registrars. Certificates are issued instantly, and registration is linked to Aadhaar and social welfare programs, reducing citizen transaction costs, improving coverage, and enhancing state efficiency.

## 4 Discussion and policy recommendations

### 4.1 Reducing the cost of recognition through health-integrated CRVS

The findings presented in the previous sections make an unequivocal case: the civil registration system in India is currently burdened with high transaction costs that discourage timely and equitable registration, especially among vulnerable populations. These costs are multidimensional—spatial, cognitive, procedural and social, which are disproportionately borne by vulnerable populations. The TCE framework allows us to view these frictions not as marginal glitches but as systemic inefficiencies stemming from suboptimal institutional arrangements (17). When the burden of transacting, i.e., completing a legal registration—outweighs the perceived or actual benefit, delay becomes a rational response rather than an act of negligence. In this context efforts must be focused more on institutional re-design that reduce transaction costs rather than awareness or reforms. The integration of health facilities into the CRVS architecture can enable timely, universal and efficient registration aligned with the goals of SDG 16.1 (1).

A core insight of TCE is that institutions function most effectively when they minimize the cost of engaging in transactions especially in uncertain, high risk or information scarce environments (10). The evidence presented in this study reinforces that health facilities, are optimally placed to serve as low-transaction-cost interfaces for civil registration owing to their geographical proximity to households, routine capture of vital events, familiarity with administrative procedures and higher level of public trust.

These features directly address the transaction cost categories identified by Williamson: search costs, negotiation costs, enforcement costs and monitoring costs (10). When an event (birth or death) occurring in a government health facility is registered at the facility, the need for families to navigate unfamiliar bureaucratic process is minimized, as the institution already holds the data, confirms the registration and completes the legal process. From an administrative point of view the registration is more reliable, timely and complete thereby freeing up resources otherwise spent on retrospective registrations, corrections and enumeration drives.

Despite this potential, in majority of the Indian states health and civil registration systems function in parallel silos, rather than as integrated governance systems. Although, medical officers authorized to issue birth certificates and institutional deliveries must trigger notifications to civil registrars, the operational effectiveness remains fragmented. Even when ANMs and ASHAs are involved in delivery support, they are not formally trained or authorized to initiate registration. Results of the study confirms that such disjuncture often leads to delays of 15–45 days, especially when families are left to navigate the process alone. From a TCE point of view this governance inefficiency can be corrected through institutional realignment.

Despite legal provisions already designating government health facilities as registrars and private institutions as informants in most Indian states, delays in birth and death registration persist due to operational gaps, unclear mandates and weak accountability (3, 27, 28). Likewise, although interdepartmental coordination committees exist at state, divisional and district levels, they remain underutilized and lack functional coherence (3). Drawing on the results of the study and to address these challenges through the lens of TCE, the following policy recommendation can reduce informational, procedural and administrative frictions:

1. *Operationalize Suo-Moto Registration in Health Facilities:* The most immediate and impactful reform is the universal adoption of *suo-moto* registration for institutional births and deaths, both in public and private sector. Under this model, registration is initiated by the health facilities using already collected information, rather than waiting for families to apply, which shifts the burden of documentation and compliance to the institutions from the individuals, thus lowering transaction costs. Health facilities already collect vital demographic information required for registration, and by integrating this information with the registration using APIs, completion of registration can be done before the family leaves the facility. This shift not only minimizes the citizen side costs but also ensure data accuracy and enables seamless linkage with schemes such as Aadhar enrolment, child nutrition, school enrolment etc.
2. *Reinforce Registrar Functions Through Role Clarity, Information Flow and Digital Integration:* Strengthening registrar functions requires clear role definitions, standardized workflows and integration between health and civil registration systems. While most public health facilities are already designated as registrars, responsibilities must be explicitly outlined, and operationalized through capacity building and work institutionalization. For private sector health institutions, legal obligations under the RBD Act and accountability mechanisms must be enforced to ensure timely reporting of vital events. Leveraging digital technology such as API integration can reduce duplication, lower the transaction costs and improve service delivery. The successful Uttar Pradesh (13, 29) pilot demonstrated that such integration reduces backend transaction costs and improves service continuity for citizens. Comprehensive registration requires engagement of all service delivery settings. Strengthening the private hospitals as reliable informants, leveraging ASHAs and ANMs to report events owing to their close community engagement can strengthen registration in India. Addressing gaps in private sector accountability and community-level reporting, alongside digital integration, is critical to ensuring every birth and death is counted. Policy measures as well as future research should focus on practical mechanisms to align these efforts into a unified, comprehensive civil registration system.
3. *Reinvigorate Interdepartmental Coordination Committees:* Co-ordination committees at the state, division and district levels must be reactivated as functional governance mechanisms with clear mandates. Regular reviews, defined clear mandates and anchoring the agenda of these units within the existing programs such as NHM, Sarva Shiksha Abhiyan (SSA) can position these bodies to function as *transaction cost reducers*—monitoring systemic delays, resolving inter-agency friction and promoting data interoperability.
4. *Embed CRVS Indicators in Health System Performance Metrics:* Integrating timely and complete birth and death registration indicators into the performance appraisals of health staff especially MOICs, ANMs and ASHAs can incentivize consistent institutional action. Facility level dashboards and performance linked incentives can improve transparency and drive competition reinforcing registration as an institutional responsibility rather than auxiliary task.
5. *Simplify Citizen Experience Through Behavioral Nudges:* Demand side transaction costs can be further minimized through behavioral interventions such as clear signage, multilingual leaflets, SMS alerts and co-location of registration facilitation counters with immunization or discharge points. Positioning registration as a gateway to child safety, school enrolment, or welfare entitlements can lead to a shift in citizen behavior much more effectively than legal coercion and this also aligns with the TCE approach of reducing cognitive and decision-making costs.
6. *Expand Death Registration Through Community-Based Tools:* Death registration especially for non-institutional and domiciliary deaths, can be improved through a combination of community-based verbal autopsy and district-level validation mechanisms, without unduly burdening the families. Frontline workers such as ASHAs and ANMs, strengthened with digital tools and clear mandate are well-positioned to play a central role in timely death registration.

The recommendation emphasis on leveraging existing health facilities, personnel and systems rather than creating new infrastructure. By leveraging countrywide health facilities, digitized data flows and coordination platforms already in place, India can make birth and death registration seamless, timely and inclusive. If scaled effectively, this approach offers a replicable model for other LMICs striving to universalize legal identity and reinforce citizen-state trust through low-friction governance.

In conclusion, delayed birth and death registration in India is not primarily a function of citizen indifference, it is a rational response to a high-friction system. Transaction costs—informational, procedural, financial and psychological are disproportionately borne by the poor, women and the rural population. The solution lies not in punitive compliance campaigns, but in institutionalizing CRVS within the health system, backed by policy clarity, workflow redesign and digital integration. Central to this reform is the practice of suo-moto registration, whereby registration is initiated proactively by designated authorities, most critically by registrars stationed at government health facilities. Suo-moto registration eliminates the need for families to initiate the process, thereby addressing the core transaction costs of search, access and procedural compliance.

When events are recorded at health facilities, those facilities not only notify the registrar but directly complete registration based on the verified occurrence of the event. Empowering the Medical Officer of a health institution, whether public or private, to initiate Suo-moto registration transforms the burden of responsibility from the citizen to the institution. This shift internalizes registration within the very system that witnesses the event, embedding legal identity creation into the existing continuum of care. In effect, registration becomes a routine output of institutional action rather than a discretionary action by citizens.

Moreover, this approach is not merely a matter of administrative convenience; it is rooted in the legal duty of the state. Under the Registration of Births and Deaths Act, every registrar is mandated to record all births and deaths occurring within their jurisdiction. For registrars based in government health facilities, this obligation is most naturally and efficiently fulfilled *in situ*, at the point of service. When the registration process is completed where the event occurs whether in a PHC, CHC, or district or private hospital it ensures fidelity, timeliness and completeness. Suo-moto registration at the facility level is therefore not only good practice but a statutory responsibility and the most viable means to meet the state’s constitutional obligation of providing legal identity to all. This shift would not only normalize registration as part of routine service delivery but also transform India’s approach to legal identity from a reactive, demand-driven model to a proactive, inclusive and efficient public good.

## 5 Conclusion

The civil registration of births and deaths is not merely a statistical obligation but a fundamental act of the state’s recognition of individual life and death, marking an individual’s entry and exit from the legal system and serving as the gateway to citizenship, rights, social protection and demographic intelligence. As the SDG goal 16.9 underscores, providing legal identity for all, including birth registration, is a moral, administrative and development imperative. However, in practice the registration remains uneven, delayed and exclusionary, especially for populations living at the margins of geography, economy and bureaucracy. The present study framed through the lens of TCE, attempts to identify the structural reasons behind this persistent gap and outline a reform path that is administratively feasible, economically rational, socially equitable and institutionally sustainable.

What emerges from the theoretical and empirical analysis is a compelling case for repositioning health facilities as the frontline institutions for civil registration. These centers are not passive providers of health services; they are embedded infrastructures with high community trust, routine data collection practices and daily contact with citizens during critical life events such as childbirth and death. The TCE framework reveals that, the current CRVS system in India externalizes the cost of registration onto the households, leading to delays, procedural confusion, information asymmetry and ultimately uneven inclusion. In contrast, embedding registration responsibilities within health facilities where the event occurs and where data already resides minimizes these transaction costs and aligns incentives across the system.

The results further provide strong empirical support for suo-moto and health facility-based registration Families delivering at government health facilities have far better chances to timely registration, while frontline workers demonstrated both willingness and capacity to support registration when digital tools and institutional authorities are in place. For suo-moto and health facility-based registration shifts the responsibility of registration from the citizens to the institutions thereby, embedding legal identity creation within the routine service delivery rather than registration being a demand driven phenomenon.

From a governance standpoint, the integration of CRVS with health systems offers several collateral benefits beyond registration completeness like improving the quality of vital statistics, reducing burden on civil registrars, fostering interdepartmental collaboration and improving the citizen’s experience of state, replacing confusion and exclusion with predictability and dignity. In doing so, it contributes to the larger goals of SDG 16, building institutions that are effective, accountable and inclusive.

India is uniquely positioned to lead this institutional transition. With its vast network of health facilities, its pioneering digital infrastructure and its experience in large-scale service delivery programs like Janani Suraksha Yojana and the National Health Mission, the country has both the tools and the momentum to achieve universal, timely and cost-efficient civil registration. The 2023 amendment to the Registration of Births and Deaths Act is a critical enabler in this direction. However, this potential requires operational integration, capacity-building and systemic realignment. The health integrated CRVS model advocated in this study is not just a policy tweak; it is a paradigm shift that treats registration not as a standalone administrative act but as an organic part of the service ecosystem that surrounds life and death.

The implications of this reform extend beyond India. Many low- and middle-income countries face similar challenges: fragmented CRVS systems, limited reach of registrars and low citizen compliance. The health-integrated model, grounded in TCE, offers a scalable and context-sensitive solution. It shifts the focus from demand-side behavioral change to supply-side institutional redesign and from episodic awareness drives to embedded service pathways. It recognizes that when transaction costs are reduced at the point of service, compliance follows naturally—not because citizens are compelled, but because the system is enabled.

In conclusion, it is worth reflecting on what is at stake. Civil registration is not just about issuing a certificate, it is about ensuring that every life is counted, every death is acknowledged and every citizen has access to the full spectrum of rights and protections that the state offers. By internalizing registration within the health systems, India can transform legal identity from a bureaucratic hurdle into a routine public good delivered at life’s most critical moments.

### 5.1 Limitation of the study

The results of the study are based on primary data collected from districts of Uttar Pradesh, which might limit the generalizability of the results. Some findings rely on self-reported experiences and therefore may be subject to recall bias and respondent interpretation.

## Data Availability

The raw data supporting the conclusions of this article will be made available by the authors, as per the ethical guidelines.

## References

[1] United Nations. Transforming our world: the 2030 agenda for sustainable development. (2015).

[2] United Nations. Principles and recommendations for a vital statistics system, revision 3. (2014).

[3] Office of Registrar General India. Vital statistics of India based on the civil registration system 2022. New Delhi: (2025).

[4] Setel PW Macfarlane SB Szreter S Mikkelsen L Jha P Stout S . A scandal of invisibility: making everyone count by counting everyone. Lancet. (2007) 370:1569–77. doi: 10.1016/S0140-6736(07)61307-5, 17992727

[5] UNICEF. Birth registration for every child by 2030: are we on track? New York, NY: (2019).

[6] World Bank and WHO. Global civil registration and vital statistics scaling up investment plan 2015–2024. (2014).

[7] Coase RH. The nature of the firm. Economica. (1937) 4:386–405. doi: 10.1111/j.1468-0335.1937.tb00002.x

[8] Williamson OE. The economics of organization: the transaction cost approach. Am J Soc. (1981) 87:548–77.

[9] Williamson OE. Markets and hierarchies: analysis and antitrust implications: a study in the economics of internal organization. New York, NY: Free Press (1975).

[10] Williamson OE. The economic institutions of capitalism. New York, NY: Free Press (1985).

[11] Burns LR Bradley EH Weiner BJ. Shortell and Kaluzny’s health care management: organization design and behavior In: Health care management. 7th ed. Boston, MA: Cengage Learning (2020)

[12] Roberts M Hsiao W Berman P Reich M. Getting health reform right. New York, NY: Oxford University Press (2008).

[13] Jain Isha. Uttar Pradesh begins online verification of birth, death certificates. The Times of India. (2022).

[14] Khan M. Now, get birth certificates at govt hospitals after delivery in Noida. Hindustan Times. (2024).

[15] PTI. No need to apply, birth certificates to be issued promptly after delivery at UP. Healthworld.com. (2023)

[16] Srivastava A. Parents Don’t need to apply for birth certificates as UP Govt implements this scheme. IndiaCom. (2023)

[17] Williamson OE. Comparative economic organization: the analysis of discrete structural alternatives. Adm Sci Q. (1991) 36:269. doi: 10.2307/2393356

[18] Meghani A Tripathi AB Bilal H Gupta S Prakash R Namasivayam V . Optimizing the health management information system in Uttar Pradesh, India: implementation insights and key learnings. Glob Health Sci Pract. (2022) 10:e2100632. doi: 10.9745/GHSP-D-21-00632, 36041830 PMC9426977

[19] Ministry of Health & Family Welfare. Health dynamics of India (infrastructure and human resources) 2022–23. New Delhi: (2024).

[20] Ministry of Health and Family Welfare Funds allocated and key achievements made under National Health Mission (2021)

[21] Ministry of Health and Family Welfare (MOHFW) Janani Suraksha Yojana (JSY) (2017)

[22] International Institute of Population Sciences (IIPS) National Family Health Survey (NFHS-5), 2019–21 (2021)

[23] International Institute of Population Sciences (IIPS). National family health survey (NFHS-4), 2015–16. India: (2017).

[24] Jaffrelot C Jumle V In: Institut Montaigne, editor. Private healthcare in India: boons and banes (2020)

[25] Office of Registrar General India The registration of births and deaths (amendment) act, 2023 (2023)

[26] Office of Registrar General India The registration of births and deaths act, 1969 (1969)

[27] Kumar K Saikia N Diamond-smith N. Performance barriers of civil registration system in Bihar: an exploratory study. PLoS One. (2022) 17:e0268832. doi: 10.1371/journal.pone.0268832, 35648782 PMC9159592

[28] United Nations. Handbook on civil registration and vital statistics systems management, operation and maintenance. (2021).

[29] Verma S. CRVS digitization in Uttar Pradesh. In: The third CR8 meeting, Dhaka, Bangladesh. Dhaka; (2023). Available online at: https://getinthepicture.org/system/files/sites/default/files/CRVS%20Digitization_0.pdf

